# Long term genitourinary toxicity following curative intent intensity-modulated radiotherapy for prostate cancer: a systematic review and meta-analysis

**DOI:** 10.1038/s41391-022-00520-x

**Published:** 2022-03-08

**Authors:** Rowan David, Alex Buckby, Arman A. Kahokehr, Jason Lee, David I. Watson, John Leung, Michael E. O’Callaghan

**Affiliations:** 1 College of Medicine and Public Health, Bedford Park, Flinders University, Bedford, SA Australia; 2 Department of Urology, SA Health, Flinders Medical Centre, Bedford, SA Australia; 3 Discipline of Medicine, Freemasons Foundation Centre for Men’s Health, University of Adelaide, Adelaide, SA Australia; 4 GenesisCare, Adelaide, SA Australia; 5 South Australian Prostate Cancer Clinical Outcomes Collaborative, Adelaide, SA Australia

**Keywords:** Prostate cancer, Urogenital diseases

## Abstract

**Background:**

Recent studies have shown that radiation-induced pelvic toxicity often requires urological consultation. However, the 10-year incidence of genitourinary toxicity following intensity-modulated radiotherapy (IMRT) amongst patients with localised prostate cancer remains unclear. Hence, we conducted a systematic review and meta-analysis to determine the incidence of late genitourinary toxicity relying on Radiation Therapy Oncology Group (RTOG) and Common Terminology Criteria for Adverse Events (CTCAE) grade as well as the incidence of specific genitourinary toxicity. Secondary objectives involved quantifing the number of studies reporting 120-month follow-up endpoints, time to event analysis, predictive factors or economic evaluation.

**Methods:**

Articles published from January 2008 to December 2021 describing prospective studies were systematically searched in MEDLINE, EMBASE and Cochrane (PROSPERO protocol CRD42019133320). Quality assessment was performed by use of the Cochrane Risk of Bias 2 Tool for RCTs and the Newcastle Ottowa Scale for non-RCTs. Meta-analysis was performed on the 60-month incidence of RTOG and CTCAE Grade ≥2 genitourinary toxicity, haematuria, urinary retention and urinary incontinence.

**Results:**

We screened 4721 studies and six studies met our inclusion criteria. All included studies involved normofractionation, three included a hypofractionation comparator arm and none involved nodal irradiation. The pooled 60-month cumulative incidence of RTOG and CTCAE Grade ≥2 genitourinary toxicity were 17% (95% CI: 5–20%, *n* = 678) and 33% (95% CI: 27–38%, *n* = 153), respectively. The pooled 60-month cumulative incidence of Haematuria was 5% (95% CI: −4–14%, *n* = 48), Urinary incontinence 12% (95% CI: 6–18%, *n* = 194), Urinary retention 24% (95% CI: 9–40%, *n* = 10). One study reported time to event analyses, one reported predictive factors, no studies reported economic analysis or 120-month toxicity. There was considerable heterogeneity amongst the studies.

**Conclusion:**

There are few high-quality studies reporting 60-month toxicity rates after IMRT. Conservative estimates of 60-month toxicity rates are high and there is need for longer follow-up and consistent toxicity reporting standards.

## Background

Recent studies have shown that patients with radiation-induced pelvic toxicity often present to urology centres for management [1, 2]. However, the incidence of genitourinary toxicity 5 to 10 years following intensity-modulated radiotherapy (IMRT), remains unclear [3–6]. The introduction of IMRT is thought to achieve a reduction in toxicity compared to Three-Dimensional Conformal Radiotherapy (3D-CRT) because of the increased treatment conformality [7, 8]. However, earlier review studies, which compared the toxicity associated with IMRT against 3D-CRT were limited by a lack of randomised prospective analyses as well as the inclusion of retrospective studies and shorter minimum follow-up periods, which may have underestimated the incidence of late genitourinary toxicity [7, 9, 10]. More high-quality studies are required to determine the late genitourinary toxicity rates because of the wide variation in radiotherapy techniques and dose regimes.

In addition, the numerous disparate late toxicity scoring systems makes interpretation of the results difficult due to lack of consistency and accuracy [7–9, 11–16]. The Radiation Therapy Oncology Group (RTOG) is one of the dominant scoring systems reported in the oncology literature, however has undergone numerous iterations to improve its accuracy. Whilst the Common Terminology Criteria for Adverse Event (CTCAE) is promoted as the comprehensive standard for reporting treatment-related adverse events in oncological care, it is often underutilised in trials. Hence the incidence of late genitourinary toxicity following IMRT remains poorly characterised [17–19].

The primary aim of this systematic review and meta-analysis was to determine the 60-month incidence of genitourinary toxicity relying on RTOG and CTCAE grade and the incidence of specific genitourinary toxicity, including haematuria, urinary retention and urinary incontinence in patients with localised prostate cancer treated with IMRT without nodal irradiation. Secondary objectives involved quantifying the number of studies reporting 120-month follow-up endpoints, time to genitourinary toxicity event analysis, predictive factors or economic evaluation.

## Methods

### Evidence acquisition

#### Selection criteria

Accepted articles were considered eligible for inclusion if they met the following criteria:

1. Population: Patients with non-metastatic biopsy-proven prostate adenocarcinoma (T1–T4, according to American Joint Committee on Cancer).
2. Intervention: Studies involving curative intent primary external beam IMRT were included. Studies that did not specify the type of radiotherapy used or included other prostate cancer treatments were excluded.
3. Comparator: A comparator group was not required because of the descriptive nature of the proposed study. However, different radiotherapy techniques, including hypofractionation and image-guided radiotherapy were considered, where reported.
4. Outcome: Late genitourinary complications after prostate radiation, as defined as 60-month following IMRT. Toxicity scoring systems that are predictive for hospitalisation, including RTOG and the CTCAE were included. The rates of haematuria, urinary incontinence and urinary retention, where available were included.
5. Study type: Prospective studies published between January 2008 and December 2021 were included. This date range was selected because it will allow comparison of outcomes associated with recent advancements in technology and dosimetry. Non English-original articles, experimental studies on animals, meeting abstracts, book chaptets, case reports and cohort studies inlving <10 patients, reviews, editorials and commentaries were not included in the review.

#### Search strategy

A comprehensive search was undertaken to systematically identify literature concerning adverse events following radiotherapy in men with prostate cancer. The following databases were searched: MEDLINE (1950—present), EMBASE (1980—present) and the Cochrane Controlled Trials Register (1991—present).

Both Medical Subject Headings (MeSH) terms and text words were used and terms common to all searches included: *prostate cancer*; *prostate carcinoma*; *prostatic neoplasms [MeSH]*; *radiation; radiotherapy; radiation injury; haematuria; bladder neck obstruction; urinary retention; urinary incontinence; erectile dysfunction*. Retrospective studies, case cohorts of <10 patients, case reports and conference abstracts were excluded. Studies only published in languages other than English were also excluded.

The review protocol, which includes the search strategy for MEDLINE, (Supplementary 1) was prospectively registered with PROSPERO (available online at https://www.crd.york.ac.uk/PROSPEROFILES/133320_STRATEGY_20220206.pdf [20] The PRISMA protocol was followed (Supplementary 2).

#### Study eligibility

The included articles from the literature search were reviewed in three consecutive phases. One researcher (RD) screened titles and abstracts for the first pass. The second pass involved a two-author (RD, AB) review of the full texts. Finally, the reference lists of the selected articles and those of previous systematic reviews were reviewed to identify other possible studies that could be included. The coding for inclusion and exclusion criteria were applied and recorded for each stage. Discrepancies were resolved with the assistance of a senior reviewer (MO’C).

#### Data extraction and analysis

Data extraction was independently performed by two authors (RD and AB) according to a preformed standardised template generated using Covidence (Veritas Health Innovation, Melbourne, Australia), an online tool for systematic reviews. We tabulated the study characteristics (author, year, country, baseline sample size, endpoint sample size, median follow-up, setting, design), patient demographics and cancer metrics (age, PSA, tumour score and grade, hormone use status, radiotherapy (fractions and dose) and secondary outcomes (60-month incidence of haematuria, urinary incontinence and urinary retention; whether the studies reported 120-month outcomes, time to event, predictive or economic analysis).

Meta-analysis was performed on the 60-month rates of RTOG or CTCAE late ≥2 genitourinary toxicity, haematuria, urinary retention and urinary incontinence using R-studio (Boston, MA 2020). A random effects model (DerSimonian–Laird method) was selected for the studies reporting genitourinary toxicity, because of the evidence of the heterogeneity in demographic and treatment characteristics amongst the studies. The *Q*-test and the *I*^2^ statistic method were performed to measure statistical heterogeneity across studies. The Chi-square test with Yates correction was used in the subgroup analysis of hypofractionation and normofractionation. Where appropriate, funnel plots were constructed to assess publication bias.

#### Quality assessment

The Cochrane Risk of Bias 2 tool was used for quality assessment for randomised controlled trials. The Newcastle Ottowa Scoring system was used to evaluate the risk of bias for non-RCT studies. The Newcastle Ottowa Scoring scores were adapted for graphical presentation by the following conversion: 2 stars = low risk, 1 star = unclear risk, 0 stars = high risk. Risk of bias analysis was performed using robvis (McGuinness, LA 2019), an online extension of an R-studio package [21].

### Evidence synthesis

#### Literature search

**Fig. 1:**
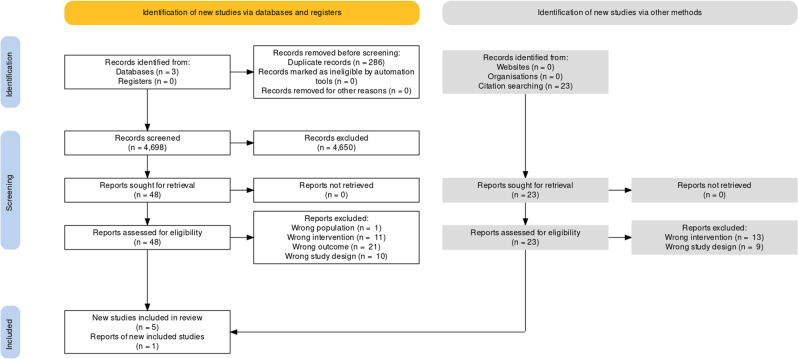
Flow diagram of evidence acquisition in a systematic review of late genitourinary toxicity in prostate cancer patients treated with IMRT.

The search yielded 4698 unique references; 4650 were excluded after reviewing the title and abstract. Of the remaining 48 studies, 43 were excluded for reasons listed in Fig. 1. One further study which was identified via citation search was included (Fig. 1). Six (0.13%) articles were included for data extraction and meta-analysis. We included one prospective cohort study [22] and five randomised control trials [23–27]. All included randomised controlled trials were phase III trials with parallel groups, of which four compared hypofractionation and normofractionation (Table 1) [24, 26, 27]. There were five multi-centre [23–27] and one single-centre study [22]. Studies were from the Netherlands [23, 24], Australia [22], France [25], Canada [27], and the UK [26].

**Table 1:** Characteristics of included studies.

Author, ref., country	Setting	Trial phase	Intervention model	Arms	Baseline sample, *N* (%)	Endpoint sample, *N* (%)	Median follow-up, months
Al-Mamgani et al. [23], Netherlands	Multi-centre	Phase III	Parallel groups	1. SIB-IMRT (78Gy/39#, no IGRT) 2. SEQ-3D-CRT (Excluded)	41 (100%)	7 (17%)	56
Sia et al. [22], Australia	Single-Centre	Prospective cohort study	Single arm	IMRT (74Gy/37#, No IGRT)	125 (100%)	32 (26%)	60
Aluwini et al. [24], Netherlands	Multi-centre	Phase III	Parallel groups	1. NFRT (78Gy/39#, mostly IGRT) 2. HYPO (64.4GY/19#)	387 (49%) 395 (51%)	97 (25%) 102 (26%)	62
Catton et al. [27], Canada	Multi-centre	Phase III	Parallel groups	1. NFRT (78Gy/39#, IGRT) 2. HYPO (60Gy/20#, IGRT)^a^	598 (50%) 608 (50%)	396 (66%) 398 (66%)	49
deCrevoisier et al. [25], France	Multi-centre	Phase III	Parallel groups	1. IGRT daily (78Gy/39#) 2. IGRT weekly (78Gy/39#)	236 (50%) 234 (50%)	437 (93%)	66
Wilson et al. [26], UK	Multi-centre	Phase III	Parallel groups	1. NFRT (74Gy/37#) 2. HYPO (60Gy/20#) 3. HYPO (57Gy/19#)	1065 (33%) 1074 (33%) 1077 (33%)	775 (24%)	72

*Gy* Greys, # Fractions *SIB* simultaneous integrated boost, *SEQ* Sequential boost, *HYPO* Hypofractionation, *NFRT* normofractionation, *IGRT* Image-guided radiotherapy, *CIMRT* Conventional fractionated intensity-modulated radiation therapy, *CIMRT* conventional fractionation intensity-modulated radiation therapy.

^a^IMRT was encouraged, although 3D-CRT was permitted in this study provided that all protocol-mandated normal tissue dose constraints were met [27].

#### Patient demographics

There was a combined total of 5840 prostate cancer patients treated with curative intent IMRT amongst the included studies. Patient demographic characteristics from the selected studies, including age, tumour stage and grade, prostate-specific antigen, hormonal status, diabetes, and cardiovascular history are summarised in Table 2. The median (range) of sample sizes at baseline was 626 (41–3216). There was a total of 2244 (38% of the baseline population) patients included at the 60-month follow-up endpoint, with sample size attrition rates ranging from 7 to 83% between studies (Table 3). Baseline IPSS was not reported in the included studies.

**Table 2:** Patient demographics from the included studies.

Author, ref.	Age	Clinical T category, *N* (%)	Gleason score, *N* (%)	PSA	ADT, *N* (%)	DM, *N* (%)	Smoking	Prostate size	Baseline IPSS	Prior TURP
Al-Mamgani et al. [23]	Mean (SD): 68.3 (6.1)	T1: 13 (32) T2: 13 (32) T3: 15 (36) T4: 0 (0)	2–4: 4 (10) 5–7: 29 (70) 8–10: 8 (20)	(Mean) 15.5	73 (41)	4 (10)	13 (32)	Not reported	Not reported	3 (8)
Sia et al. [22]	Median: 69	T1: 25 (20) T2: 57 (45) T3: 37 (30) T4: 6 (5)	2–6: 40 (32) 7: 60 (48) 8–10: 25 (20)	<10: 35 (28) 10–20: 42 (34) >20: 48 (38)	120 (96)	Not reported	Not reported	Not reported	Not reported	Not reported
Aluwini et al. [24]	Median: 70	T1: 113 (14) T2: 263 (34) T3: 397 (51) T4: 9 (1)	6: 238 (30) 7: 355 (45) 8: 115 (15) 9: 67 (9) 10: 7 (1)	(Median) 14	519 (66)	Not reported	Not reported	>50 cm^3^ 25% (HYPO), 25% (NFRT)	Not reported	75 (10)
Catton et al. [27]	Median: 72	T1: 636 (53) T2: 560 (47) T3: 0 T4: 0	3 + 3: 113 (9) 3 + 4: 762 (63) 4 + 3: 331 (28)	<5: 219 (18) 5–10: 605 (50) 10.1–20: 382 (32)	68 (6)	Not reported	Not reported	Not reported	Not reported	Not reported
deCrevoisier et al. [25]	Median: 70	T1: 205 (44) T2: 112 (24) T3: 153 (33) T4: 0	4–6: 124 (26) 7: 303 (64) 8–10: 43 (9)	(Median) 11	219 (47)	51	Not reported	Not reported	Not reported	Not reported
Wilson et al. [26]	<75: 2725 =/>75: 491	T1: 1170 (37) T2: 1756 (56) T3: 227 (7) T4: 0 (0)	≤6:1122 (35) 7: 1995 (62) 8: 99 (3)	(Mean) 11	3126 (97)	342	Not reported	Median 37 (<75 years)/42.7 (≥75 years)	Not reported	259 (8)

*PSA* Prostate-specific antigen, *ADT* Androgen deprivation therapy, *DM* Diabetes mellitus, *FU* Follow-up, *HYPO* Hypofractionation, *NFRT* Normofractionation.

**Table 3:** Secondary outcomes.

Study	Haematuria, *N* (%)	Urinary incontinence, *N* (%)	Urinary retention, *N* (%)	120-month endpoint	Time to event	Predictive factors	Economic analysis
Al-Mamgani et al. [23]	0 (0)	2 (6)	10 (24)	No	No	No	No
Aluwini et al. [24]	No	127 (16)	No	No	No	Yes	No
Wilson et al. [26]	No	No	No	No	Yes	No	No
deCrevoisier et al. [25]	4 7(10)	65 (14)	No	No	No	No	No
Sia et al. [22]	1 (1.25)	No	No	No	No	No	No
Catton et al. [27]	No	No	No	No	No	No	No

#### Incidence of late genitourinary toxicity

**Fig. 2:**
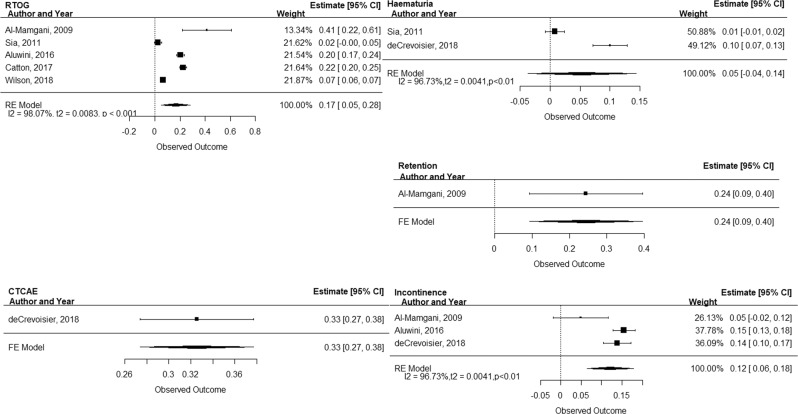
Forrest plots of studies included in the meta-analysis demonstrating the 60-month incidence of RTOG and CTCAE ≥ 2 toxicity, haematuria, urinary incontinence and retention.

Five studies reported toxicity with the RTOG scale [22–24, 26]. The pooled 60-month RTOG ≥ 2 genitourinary toxicity incidence was 17% (95% CI: 5–28%) based on a random effects model (*I*^2^ 98%; Fig. 2). The one included study that reported CTCAE ≥ 2 genitourinary toxicity demonstrated a 60-month incidence rate estimate of 33% (95% CI: 27–38%) based on a fixed-effects model (Fig. 2) [25].

#### Incidence of specific genitourinary toxicity

Three studies reported the rate of haematuria at a 60-month endpoint with a pooled 60-month incidence rate estimate of 5% (95% CI: −4–14%), based on a random effects model (*I*^2^ 96.73%; Fig. 2) [22, 23, 25]. Three (60%) studies reported urinary incontinence at 60-month follow-up endpoint, with a pooled 60-month incidence rate estimate of 12% (95% CI: 6–18%), based on a random effects model (Fig. 2) [23–25]. One (20%) study reported urinary retention at 60 months, with a 60-month incidence rate estimate of 24% (95% CI: 9–40%), based on a fixed-effects model (Fig. 2) [23]. One study reported time to event analysis amd [26] one reported predictive factors analysis [24]. None of the included studies included economic analysis (Table 3).

#### Subgroup analysis

**Fig. 3:**
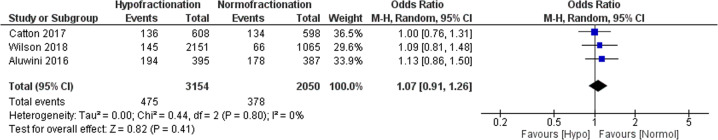
Forrest plots of studies included in the meta-analysis comparing the 60-month incidence of RTOG amongst patients treated with hypofractionation and normofractionation intensity-modulated radiotherapy.

Three of the included studies compared men with localised prostate cancer treated with either hypofractionated or normofractionated intensity-modulated radiotherapy [24, 26, 27]. All three of these studies reported RTOG genitourinary toxicity, with RTOG ≥ 2 late genitourinary toxicity occurring in 475/3154 (15%) and 378/2050 (18%) of the hypofractionation and normofractionation arms, respectively. There was no significantly significant difference in RTOG Grade ≥2 genitourinary toxicity at 60 months post-radiotherapy amongst men with localised prostate cancer treated with normofractionation compared with hypofractionation (1.07, 95% CI: 0.91, 1.26, *p* = 0.41), based on a random effects model (Fig. 3) [24, 26, 27].

#### Risk of bias assessment

**Fig. 4:**
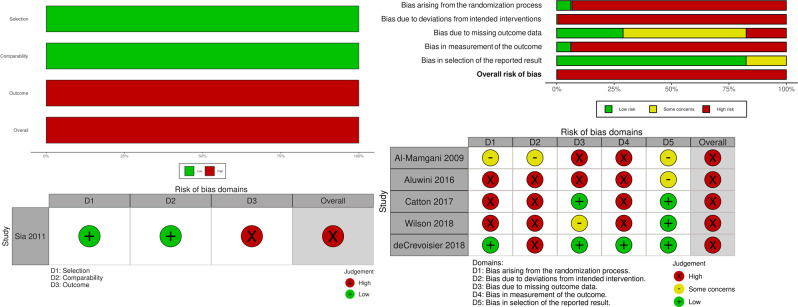
Weighted summary bar plot and traffic light plot for included RCTs and non-RCT based on the Cochrane Risk of Bias 2 Tool and Newcastle Ottowa Score, respectively.

Weighted summary bar plots of the studies assessing the incidence rate of late genitourinary toxicity revealed an overall high risk of bias for all studies based on the Cochrane Risk of Bias 2 Tool. A large proportion of the bias was due to the lack of blinding of participants and outcome assessors (Fig. 4). For each analysis, there were less than ten included studies, reducing the usefulness of funnel plot presentations to assess publication bias.

## Discussion

**Fig. 5:**
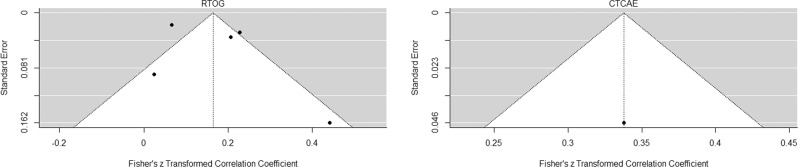
Funnel plots of heterogeneity for studies included for meta-analysis of late RTOG and CTCAE genitourinary toxicity rates.

Our systematic literature review of prospective studies reporting long-term urologic complications after radiation therapy treatment for prostate cancer included five articles in a meta-analysis, with a pooled RTOG ≥ 2 incidence of 17% (95% CI: 5–20%). Additionally, the single study included that assessed late CTCAE grade ≥2 genitourinary toxicity reported a 33% incidence (95% CI: 27–38%). These two metrics correlate well, with a reported 10% under-estimation of toxicity as measured by RTOG compared with CTCAE [28]. Our meta-analysis revealed a strong effect size with broad confidence intervals and considerable heterogeneity amongst studies Fig. 5. Overall, the toxicity rates reported likely remain a conservative estimate given under reporting and bias due to lack of blinding in those assessing the outcomes.

This study reports a 5% (95% CI: −4 to 14%) pooled incidence rate estimate of haematuria at 60 months post-IMRT, which is consistent with rates reported elsewhere in the literature [29–31]. The incidence of radiation cystitis remains controversial, with reported estimates ranging from 2.6 to 12.1% amongst mostly low-level evidence studies including retrospective series and conference abstracts, which often lack documentation of toxicity diagnosis and reporting of validated toxicity scoring systems [29, 30, 32]. The current study reports 12% (95% CI: 6–18%) and 24% (95% CI: 9–40%) pooled 60-month rate estimates of urinary incontinence and urinary retention, respectively. Unfortunately, the rate of urinary retention was only reported in one of the included studies, which had a very small sample size (*n* = 41 at baseline, *n* = 7 at 5 years post treatment and n = 10 with urinary retention) and is likely overestimated [23]. The need for long-term follow-up of lower urinary tract symptoms was highlighted by the recent meta-analysis by Awad et al. [33] which found that an increase in median follow-up time after prostate EBRT led to a significantly increased risk of developing urethral strictures (OR 0.005, 95% CI 0.0002–0.01, *p* = 0.041). The predictive factors of radiation-induced genitourinary complications remain unclear. Currently, the literature consists of observational studies of radiotherapy complications but lacks review studies grouping the data. The cost associated with radiation therapy-related complications also remains poorly described, despite the growing number of global economic comparative evaluations of treatments for localised prostate cancer [19, 34, 35]. Furthermore, the cumulative incidence of treatment-related genitourinary at 120 months was unable to be determined due to lack of reporting in the included trials and may be higher and more severe, given the progressive fibrosis that can develop in patients with radiotherapy-related toxicity [29]. Other recent meta-analyses have also shown no statistically significant differences in late genitourinary toxicity amongst men with prostate cancer treated with hypofractionated radiotherapy compared with conventional radiotherapy [11, 36].

The current study has several limitations, including a small number of included studies, high heterogeneity between studies and predominant use of the RTOG system, which may miss complications. The meta-analysis was dominated by the inclusion of 3216 (69%) patients from the CHHip trial [26], with the main dose fractionation schedule of 74Gy/37#, which is now outdated. Furthermore, radiotherapy in the CHHiP trial was not routinely delivered with image-guidance and involved larger margins than typically expected [26]. Similarly, most of the included studies use generous margins with unclear standards for IGRT [22–24, 27]. In addition, the PROFIT trial by Catton et al. included an unreported proportion of patients treated with 3D-CRT who met the protocol-mandated normal tissue dose constraints [27]. Some relevant trials may have been excluded as they did not meet the inclusion criteria [37–39]. However, the vast majority of these studies were low-level single institution retrospective studies, which are likely to underestimate toxicity given the reliance on physician reported rather than patient reported outcomes. Furthermore, the included studies involved contemporary radiotherapy techniques, and were all prospective and mainly RCTs, with standardised outcome measurements.

This study reports the incidence of complications but does not differentiate toxicity grades or compare to alternative treatment pathways (e.g. radical prostatectomy), as the data was not provided in the included studies. Furthermore, this study does not evaluate the long-term toxicity associated with adjuvant or salvage radiotherapy, which exposes larger portions of adjacent normal tissue to radiotherapy, and which is likely also underreported. This study does not include an exhaustive assessment of genitourinary toxicity and omits quality of life outcomes which may be equally important [40–44]. Whilst the pooled incidence rate is likely an underestimate in aggregate, it may also be an overestimate for patients with a small prostate and low baseline IPSS and those treated with IGRT. While we report a correlation between radiotherapy treatment and the development of symptoms such as haematuria, urinary incontinence and retention over 60 months this association may not be causal. There is a need for a prospective population-level dataset with central registration for patients with confirmed late radiation cystitis, urinary tract strictures and necrotic bladder neck contractures to allow for baseline assessment and formal standardisation.

## Conclusions

The current study presents the first consolidated literature review and meta-analysis on long-term genitourinary outcomes in patients with prostate cancer treated with primary IMRT. The 60-month incidence of genitourinary toxicity following IMRT provided in the current study exceeds traditional expectations and is likely a conservative estimate. Furthermore, the paucity of high-quality studies reporting late toxicity is concerning. Future studies of radiotherapy techniques should involve longer follow-up and improved toxicity reporting standards.

## Supplementary information

Supplementary 1

Supplementary 2
