# Supplementary material for: Long term genitourinary toxicity following curative intent intensity-modulated radiotherapy for prostate cancer: a systematic review and meta-analysis
Source: Prostate Cancer Prostatic Dis. 2022 Mar 8;26(1):8–15. doi: 10.1038/s41391-022-00520-x (PMC10023565; doi:10.1038/s41391-022-00520-x)
Supplement: Supplementary file 1 — Supplementary 1 [file 41391_2022_520_MOESM1_ESM.pdf]

Supplementary 1

Search strategy for MEDLINE

Database(s): **Ovid MEDLINE(R) and Epub Ahead of Print, In-Process, In-Data-Review & Other Non-Indexed Citations, Daily and Versions(R)** 1946 to February 04, 2022
Search Strategy:

#	Searches	Results
1	exp Prostatic Neoplasms/	139475
2	(Prostat* adj2 (neoplasm* or cancer* or adenocarcinom* or mass or tumor)).tw,kf.	144866
3	or/1-2	177067
4	exp radiotherapy/ or exp brachytherapy/ or exp proton therapy/ or exp radiosurgery/ or exp radiotherapy, image-guided/ or exp x-ray therapy/	199125
5	(external beam radiotherap* or radiation therap* or radiotherap* or external beam* radiation therap* or EBRT).tw,kf.	265953
6	(brachytherapy* or curietherapy* or (radiotherap* adj3 implant*) or ((intracavit* or interstitial) adj3 radiotherap*) or ((radioisotope* or "radio isotope") adj3 therap*)).tw,kf.	20895
7	(SBRT or radiosurger* or (stereotactic adj3 bod adj3 radiotherap*) or (stereotactic adj3 bod adj3 radiation therap*)).tw,kf.	19006
8	(IMRT or (Intensity adj3 modulated adj3 radiotherap*) or (Intensity adj3 modulated adj3 radiation therap*) or (volumetric-modulated adj3 Arc adj3 Therap*) or (Intensity-modulated adj3 arc adj3 therap*) or (volumetric modulated adj3 Arc adj3 Therap*) or (Intensity modulated adj3 arc adj3 therap*) or (helical adj3 tomotherap*)).tw,kf.	16734
9	(IGRT or ((image-guided or (image adj3 guided)) adj3 (radiotherap* or radiation therap*)) or (target organ adj3 (alignment or alinment) adj3 (radiotherap* or radiation therap*))).tw,kf.	3555
10	or/4-9	365075
11	Hematuria/ or haematuria.tw,kf.	15299
12	Radiation Injuries/ or radiation cystitis.tw,kf.	35207
13	bladder neck obstruction.tw,kf. or Urinary Bladder Neck Obstruction/	4743
14	urethral stricture.tw,kf. or Urethral Stricture/	6848
15	urinary retention.tw,kf. or Urinary Retention/	11837
16	urinary incontinence.tw,kf. or Urinary Incontinence/	38900
17	erectile dysfunction.tw,kf. or Erectile Dysfunction/	26275
18	or/11-17	133426
19	3 and 10 and 18	3154
20	human/	20150552

21	(human or male or men or man).mp.	12169833
22	20 or 21	22495199
23	19 and 22	3076
24	limit 23 to (english language and yr="2008 -2021")	1803
25	(letter or editorial or note or commentary).pt.	1763817
26	24 not 25	1738
